# Awareness and use of e-cigarettes among university students in Shanghai, China

**DOI:** 10.18332/tid/125748

**Published:** 2020-09-08

**Authors:** Wenyuanyue Wang, Maojie Lu, Yuyang Cai, Nannan Feng

**Affiliations:** 1 School of Public Health, Shanghai Jiao Tong University School of Medicine, Shanghai, China; 2 China Institute for Urban Governance, Shanghai Jiao Tong University, Shanghai, China

**Keywords:** prevalence, awareness, university students, perception, e-cigarettes

## Abstract

**INTRODUCTION:**

The use of electronic cigarettes (e-cigarettes) in adults is increasing globally, and similar trends may be observed in the young population. Our objective was to estimate the awareness and use of e-cigarettes among the students from two comprehensive universities in Shanghai, China, and to identify the factors that may influence their decision to use e-cigarettes and their possible adverse effects.

**METHODS:**

An online cross-sectional survey was conducted among the students of Shanghai Jiao Tong University and Fudan University. A total of 869 students (412 males and 457 females), mean age 21.09 years (SD=2.44), were recruited in 2018. Multivariate binary logistic regression analyses were performed to explore the associations between ever e-cigarette use and influencing factors.

**RESULTS:**

Of the responding students, 88.4% were aware of e-cigarettes, 4.6% had used e-cigarettes at least once in their lifetime, and 1.7% were current e-cigarettes users. Males and smokers were more likely to use e-cigarettes (ever used e-cigarettes even once) than females (OR=3.51; 95% CI: 1.69–7.27; p=0.001) and non-smokers (OR=28.58; 95% CI: 14.03–58.20; p<0.001). University students were easily motivated to use e-cigarettes when their peers also used them, and the risk ratio was 4.15 (95% CI: 2.11–8.19) compared with if their peers never used e-cigarettes. The major factors found to motivate university students to use e-cigarettes were the belief that e-cigarettes were less harmful or not harmful (55.0%) and the perception that e-cigarettes were helpful to quit smoking (37.5%). The survey also indicated that 72.4% of the respondents heard about e-cigarettes from television advertisements, 42.7% from websites online, and 41.2% from their parents and friends.

**CONCLUSIONS:**

University students who were males, cigarette smokers and whose peers used e-cigarettes were more likely to use e-cigarettes. The use of traditional cigarettes should be controlled strictly in order to reduce the likelihood of e-cigarette use among university students.

## INTRODUCTION

Electronic cigarettes (e-cigarettes), also known as electronic nicotine delivery systems, are hand-sized battery-operated devices, which were originally designed to retain the look and feel of traditional cigarettes by delivering nicotine vapor to the user^1^. E-cigarettes consist of a power source, a heating element, and a tank or cartridge containing an ‘e-liquid’ in which there are nicotine (or no nicotine) and flavors dissolved in glycerol-propylene glycol^2^. Inhaling nicotine through e-cigarettes without combustion is believed to be safer than traditional tobacco burning by eliminating harmful combustion products, including tar and carbon monoxide^3^.

The device was first introduced to the Chinese domestic market in 2003 as an aid for smoking cessation and a replacement for regular cigarettes^4^. The International Tobacco Control (ITC) China survey showed that the percentage of smokers who had heard of e-cigarettes increased from 29.0% to 60.0%, and those who had tried e-cigarettes from 2.0% to 11.0%, in the period 2009 to 2014^5^. According to the China Adult Tobacco Survey 2015, 52.3% of the population aged 15–24 years were aware of e-cigarettes and 4.1% had tried e-cigarettes, which were both higher than in other age groups^6^. In 2015, one mobile app-based survey in China, which recruited 2042 adolescents aged 12–18 years, showed that 89.5% of the surveyed adolescents were aware of e-cigarettes and 26.4% of the respondents had used e-cigarettes^7^. But we inferred the numbers might be higher than the true figures since people who were interested in e-cigarettes were more likely to participate in the online questionnaire. Another report indicated that 45.0% of middle school students had heard of e-cigarettes and 1.2% of participants had used e-cigarettes in the last 30 days^8^. A cross-sectional study including 5482 university students in Texas indicated that perceptions of harm and addictiveness of e-cigarettes were lower than those for conventional cigarettes among university students^9^. Furthermore, young adults are more likely to be aware of and use e-cigarettes if current trends in e-cigarette advertising continue^10^; common e-cigarette advertising is designed to imply safety and smoking cessation, and incorporates health claims not supported by medical evidence^11,12^.

In China, the lack of regulations for e-cigarette use and the unrestricted practice^13,14^ encouraged the increase in the adoption of e-cigarettes and the misconceptions of the benefits of using e-cigarettes, especially among young adults^12^, since e-cigarettes are marketed to appeal to youth^14,15^. However, few studies have examined awareness and use of e-cigarettes among Chinese university students and the predictors of e-cigarette use, which are crucial to understanding the use of e-cigarettes by college students and the associated motivational factors. Therefore, our objective was to estimate the awareness and use of e-cigarettes among university students in Shanghai and to explore the possible predictors that encourage e-cigarettes use.

## METHODS

### Participants and procedure

A cross-sectional survey was developed among undergraduates and graduate students who studied in Shanghai Jiao Tong University and Fudan University, two top universities in Shanghai, in the summer semester of 2018. The survey questionnaire was designed based on the standard Chinese urban adult tobacco questionnaire and the network survey^16,17^. We adjusted parts of the questionnaire according to the investigated subjects and our experience, including the baseline information and the cognition on the health hazards of e-cigarettes. The survey was made available in these two universities through an application called ‘So Jump’ which can design, distribute and recover questionnaires online, between March and April 2018. Taking a class as the unit, we used the cluster sampling to collect information about awareness and use of e-cigarettes. The survey was completely anonymous. In total, 1046 participants were selected, while 869 university students (including 813 non-smokers who had never tried traditional cigarettes, and 56 smokers who had smoked) completed the questionnaire with a response rate of 83.1%. Ethical approval was not necessary according to the Ethics Committee of School of Public Health, Shanghai Jiao Tong University School of Medicine, as this study was not an experimental study.

### Measures

We gave the questionnaire, named ‘Survey on the Awareness and Use of E-cigarettes’, to the college counselors who distributed it to the entire class of students through the ‘So Jump’ application.

The respondents were first asked about their age, gender, specialization and smoking experience (categorized as smokers and non-smokers). Participants were divided into three groups according to grade: junior students, senior students, and graduate students. Next, we assessed their awareness of e-cigarettes through the question: ‘Have you ever heard of e-cigarettes?’. Participants who answered ‘Yes’ were further asked: ‘Where have you heard of e-cigarettes?’ (multiple choice). All respondents were asked about their perceptions of e-cigarettes. The first question in this part was about student’s general perspective of e-cigarettes. The choices were: ‘Smoking cessation products’, ‘Environmentally friendly (producing less harmful substance to environment) alternatives to traditional cigarettes’, and ‘Electronic product’. The students then answered several questions about health hazards, addiction, and smoking cessation help of e-cigarettes. Students who thought e-cigarettes were environmentally friendly alternatives to traditional cigarettes, healthier and less addictive than traditional cigarettes were considered to have a positive opinion of e-cigarettes. Those who thought e-cigarettes could help quit smoking and avoid secondhand smoke, and contained no carcinogens, were also considered to have a positive opinion of e-cigarettes.

Respondents who answered ‘Yes’ to the question ‘Do you vape e-cigarettes currently?’ were categorized as current e-cigarette users and those who answered ‘Yes’ to ‘Have you ever tried e-cigarettes (even once)?’ were categorized as ever e-cigarette users. Finally, we asked students whether there was anyone in their social circle that used e-cigarettes and whether they experienced any adverse effects from e-cigarettes.

The final version of our questionnaire comprised 23 questions, 19 of which were single-choice questions and 7 questions that provided a ‘not sure’ option. The questionnaire is given in the Supplementary file.

### Statistical analysis

Data were analyzed using SPSS version 22.0 and were verified for normality of distribution and equality of variances. The quantitative variables were presented as mean and standard deviation (±SD) and qualitative data were described as frequency and percentage (%). Chi-squared tests were performed to compare awareness ratio of e-cigarettes and prevalence of ever use by demographics and other basic information. We then conducted unadjusted and multivariate logistic regression analyses to explore the risk factors of ever e-cigarette use, with an odds ratio calculation. In multivariate logistic regression analyses, ever e-cigarettes use was a dependent variable. Independent variables included demographics and variables identified by unadjusted logistic regression analysis that had a statistically significant association with ever e-cigarette use. Significant test was a bilateral test and the level of statistical significance was set at p<0.05 for all the analyses.

## RESULTS

### Characteristics of participants

In all, 869 participants out of 1046 (83.1%) completed all survey questions. Among the valid respondents, 412 (47.4%) were males and 457 (52.6%) were females, with an average age of 21.09±2.44 years. There were 235 (27.0%) junior students, 398 (45.8%) senior students, and 236 (27.2%) graduate students. As for their specialization, the majority of respondents (44.4%) were medical students, 24.3% were engineering students, 23.1% were science students, and 8.2% were liberal art students. In total, 56 (6.4%) respondents reported cigarette use, with median years of smoking 2 years (IQR:1–4 years).

### Awareness of e-cigarettes

Respondents who answered ‘yes’ to the question ‘Have you ever heard of e-cigarettes?’ were considered to be aware of e-cigarettes. In our survey, the majority (88.4%) of participants were aware of e-cigarettes (88.6% of men and 88.2% of women, with no statistical difference). Of the graduate students, 92.8% were aware of e-cigarettes, while 88.4% of the senior students and 83.8% of the junior students were aware of them, with a significant difference (p=0.01). Of the medical students, 92.7% were aware of e-cigarettes, which was higher than that of the engineering students (83.9%) and science students (84.6%) (p=0.003). Cigarette smokers (96.4%) were more aware of e-cigarettes than never smokers (87.5%) in our study (p=0.004) (Table 1).

**Table 1:** Awareness of e-cigarettes among university students, Shanghai, China 2018 (N=869)

	*Total*	*Have you ever heard of e-cigarettes?*		*p*
	*n*	*Yes n (%)*	*No n (%)*	
**All**	869	768 (88.4)	101 (11.6)	
**Gender**				
Male	412	365 (88.6)	47 (11.4)	**0.851**
Female	457	403 (88.2)	54 (11.8)	
**Grade**				
Junior	235	197 (83.8)	38 (16.2)	**0.010**
Senior	398	352 (88.4)	46 (11.6)	
Graduate	236	219 (92.8)	17 (7.2)	
**Specialization**				
Medical	386	358 (92.7)	28 (7.3)	**0.003**
Engineering	211	177 (83.9)	34 (16.1)	
Science	201	170 (84.6)	31 (15.4)	
Liberal art	71	63 (88.7)	8 (11.3)	
**Cigarette use**				
Yes	813	784 (96.4)	29 (3.6)	**0.004**
No	56	49 (87.5)	7 (12.5)	

The majority (72.4%) of students heard about e-cigarettes from television advertisements and about half of the students (42.7%) knew about e-cigarettes through the internet. Elsewhere, university students knew about e-cigarettes directly through their parents, friends and classmates (41.2%), or indirectly from other ways (27.7%) such as a micro-blog and Wechat (the most frequently used communication and social media applications in China, similar to Facebook and Twitter), from stores and supermarkets (11.0%), pharmacies (2.5%) and other channels (2.4%) (Supplemental file, Tables S1 to S4).

### Perception of e-cigarettes

The majority of participants had a positive opinion of e-cigarettes. Most respondents thought that e-cigarettes were healthier (78.0%) and less addictive (63.1%) than regular cigarettes. Most (72.6%) declared that e-cigarettes were used as an environmentally friendly alternative to traditional cigarettes and 81.0% thought that e-cigarettes were helpful for quitting smoking. More than half of the respondents reported that e-cigarettes could help avoid secondhand smoke and contained no carcinogens or chose ‘not sure’. Furthermore, they believed that e-cigarettes would replace traditional cigarettes partly or completely in the future (70.9%) (Table 2).

**Table 2:** Perception of e-cigarettes among university students, Shanghai, China 2018 (N=869)

*Items*	*Frequency n*	*Percentage %*
**What kind of products are e-cigarettes?**		
Smoking cessation products	495	57.0
Environmentally friendly alternatives to traditional cigarettes	631	72.6
Electronic products	141	16.2
**Can e-cigarettes help quit smoking?**		
Completely effective	18	2.1
Helping relieve abstinence symptoms	193	22.2
Helping reduce smoking	493	56.7
Completely invalid	165	19.0
**Can e-cigarettes replace traditional cigarettes?**		
Completely	13	1.5
Partly	603	69.4
No	83	9.6
Not sure	170	19.6
**Do you think teenagers are more interested in e-cigarettes?**		
Yes	310	35.7
No	329	37.9
Not sure	230	26.5
**Health hazards of e-cigarettes compared with traditional cigarettes**		
Completely healthy	74	8.5
Less harmful	604	69.5
Equally	70	8.1
Not sure	121	13.9
**Addiction of e-cigarettes compared with traditional cigarettes**		
Stronger	13	1.5
Equally	133	15.3
Weaker	443	51.0
No addiction	105	12.1
Not sure	175	20.1
**Do you think e-cigarettes could avoid harm of secondhand smoke?**		
Yes	302	34.8
No	314	36.1
Not sure	253	29.1
**Do you think e-cigarettes have carcinogens?**		
Yes	188	21.6
No	301	34.6
Not sure	380	43.7

We also addressed how perception of e-cigarettes may be influenced and found that men and junior students were more likely to perceive e-cigarettes as being less harmful than cigarettes and more helpful in quitting smoking (all p<0.05). Interestingly, smokers were more likely to perceive e-cigarettes as not helpful for quitting smoking compared with never smokers (p=0.009). Men (p<0.001) and senior students (p<0.001) were more inclined to believe that e-cigarettes could help avoid secondhand smoke. In addition, men, junior and senior students, and non-medical students, were more likely to perceive e-cigarettes as having no carcinogens (all p<0.001) (Supplemental file, Tables S1 to S4).

### Use of e-cigarettes

Only 4.6% of participants reported that they had ever used e-cigarettes, 1.7% of participants were current e-cigarette users and two people (0.2%) had been using e-cigarettes every day. The ever e-cigarette users reported various reasons for their behavior such as ‘less harmful than traditional cigarettes’ (55.0%), ‘helpful for quitting smoking’ (37.5%), ‘diverse flavors’ (27.5%), ‘avoiding secondhand smoke’ (22.5%), ‘fashionable’ (17.5%), ‘can be used in smoke-free places’ (12.5%), and ‘alternatives to traditional cigarettes (10.0%)’.

We also established the predictors of ever e-cigarette use by unadjusted and multivariate logistic regression analyses (Table 3). Unadjusted logistic regression analysis found that men (7.3%) were more likely to use e-cigarettes (ever used e-cigarettes even once) than women (2.2%) (OR=3.51; 95% CI: 1.69–7.27; p=0.001). We also found that non-medical students were more likely to use e-cigarettes than medical students (OR=3.35; 95% CI: 1.53–7.36; p=0.003). The cigarette smokers (39.3%) were more likely to use e-cigarettes than never smokers (2.2%) (OR=28.58; 95% CI: 14.03–58.20; p<0.001). The students whose peers used e-cigarettes were more likely to use e-cigarettes than those with no peers using them (OR=4.15; 95% CI: 2.11–8.19; p<0.001). Moreover, we found that ever e-cigarette use was highly correlated with perceptions of e-cigarettes such as: teenagers are no more interested in e-cigarettes (OR=0.39; 95% CI: 0.19–0.82; p=0.012) or not sure (OR=0.20; 95% CI: 0.07–0.59; p=0.003), e-cigarettes can help to reduce smoking (OR=0.33; 95% CI: 0.16–0.67; p=0.002) or help to relieve abstinence symptoms (OR=0.30; 95% CI: 0.11–0.78; p=0.014) or help to quit smoking (OR=0.55; 95% CI: 0.07–4.39; p=0.571), e-cigarettes will replace the regular cigarettes partially (OR=0.25; 95% CI: 0.05–1.18; p=0.079) or e-cigarettes will not replace the regular cigarettes (OR=0.59; 95% CI: 0.11–3.13; p=0.532) or not sure (OR=0.13; 95% CI: 0.02–0.81; p=0.028).

**Table 3:** Unadjusted and Multivariate logistic regression analyses of factors that affect ever e-cigarettes use, Shanghai, China 2018 (N=869)

*Variables*	*Ever e-cigarette use*		*Unadjusted*		Multivariate*	
	*Yes n (%)*	*No n (%)*	*OR (95% CI)*	*p*	*AOR (95% CI)*	*p*
Age			0.961 (0.837–1.103)	0.568	0.995 (0.835–1.185)	0.954
**Gender**				**0.001**		0.214
Female	10 (25.0)	447 (53.9)	1		1	
Male	30 (75.0)	382 (46.1)	3.510 (1.694–7.274)		1.743 (0.725–4.188)	
**Grades**				0.301		
Junior/Senior	32 (80.0)	601 (72.5)	1			
Graduate	8 (20.0)	228 (27.5)	0.659 (0.299–1.451)			
**Specialization**				**0.003**		0.380
Medical	8 (20.0)	378 (45.6)	1		1	
Non-Medical	32 (80.0)	451 (54.4)	3.353 (1.527–7.363)		1.537 (0.589–4.012)	
**Traditional cigarettes use**				**<0.001**		**<0.001**
No	18 (45.0)	795 (95.9)	1		1	
Yes	22 (55.0)	34 (4.1)	28.578 (14.033–58.200)		16.651 (7.100–39.048)	
**Use of e-cigarettes in social circle**				**<0.001**		**0.008**
No	18 (45.0)	594 (71.7)	1		1	
Yes	18 (45.0)	143 (17.2)	4.154 (2.108–8.186)	<0.001	3.718 (1.588–8.704)	0.002
Not sure	4 (10.0)	92 (11.1)	1.435 (0.475–4.334)	0.522	1.123 (0.311–4.053)	0.859
**Can e-cigarettes help quit smoking?**				**0.011**		0.200
Completely invalid	16 (40.0)	149 (18.0)	1		1	
Helping reduce smoking	17 (42.5)	476 (57.4)	0.333 (0.164–0.674)	0.002	0.359 (0.136–0.949)	0.039
Helping relieve abstinence symptoms	6 (15.0)	187 (22.6)	0.299 (0.114–0.782)	0.014	0.438 (0.130–1.478)	0.183
Completely effective	1 (2.5)	17 (2.1)	0.548 (0.068–4.392)	0.571	0.920 (0.088–9.584)	0.944
**Can e-cigarettes replace traditional cigarettes?**				**0.027**		0.773
Completely	2 (5.0)	11 (1.3)	1		1	
Partly	16 (65.0)	577 (69.6)	0.248 (0.052–1.176)	0.079	0.654 (0.088–4.847)	0.678
No	8 (20.0)	75 (9.0)	0.587 (0.110–3.128)	0.532	1.198 (0.132–10.864)	0.872
Not sure	4 (10.0)	166 (20.0)	0.133 (0.022–0.805)	0.028	0.853 (0.086–8.472)	0.892
**Do you think teenagers are more interested in e-cigarettes?**				**0.002**		0.079
Yes	25 (62.5)	285 (34.4)	1		1	
No	11 (27.5)	318 (38.4)	0.394 (0.191–0.816)	0.012	0.472 (0.191–1.169)	0.105
Not sure	4 (10.0)	226 (27.3)	0.202 (0.069–0.588)	0.003	0.260 (0.068–0.997)	0.049
**Health hazards of e-cigarettes compared with traditional cigarettes**				0.251		
Completely healthy	7 (17.5)	67 (8.1)	1			
Less harmful	25 (62.5)	579 (69.8)	0.413 (0.172–0.992)	0.048		
Equally	3 (7.5)	67 (8.1)	0.429 (0.106–1.728)	0.234		
Not sure	5 (12.5)	116 (14.0)	0.413 (0.126–1.351)	0.144		
**Addiction of e-cigarettes compared with traditional cigarettes**				**0.005**		0.259
Stronger	1 (2.5)	12 (1.4)	1		1	
Equally	11 (27.5)	122 (14.7)	1.082 (0.128–9.116)	0.942	0.459 (0.044–4.794)	0.516
Weaker	16 (40.0)	427 (51.5)	0.450 (0.055–3.672)	0.456	0.303 (0.029–3.167)	0.319
No addiction	11 (27.5)	94 (11.3)	1.404 (0.166–11.858)	0.755	0.367 (0.033–4.053)	0.413
Not sure	1 (2.5)	174 (21.0)	0.069 (0.004–1.172)	0.064	0.041 (0.002–0.958)	0.047
**Do you think e-cigarettes could avoid harm of secondhand smoke?**				0.386		
Yes	17 (42.5)	285 (34.4)	1			
No	15 (37.5)	299 (36.1)	0.841 (0.412–1.716)	0.634		
Not sure	8 (20.0)	245 (29.6)	0.547 (0.232–1.290)	0.168		
**Do you think e-cigarettes have carcinogens?**				0.597	
Yes	11 (27.5)	177 (21.4)	1			
No	14 (35.0)	287 (34.6)	0.785 (0.349–1.767)	0.559		
Not sure	15 (37.5)	365 (44.0)	0.661 (0.298–1.469)	0.310		

* AOR: adjusted odds ratio; adjusted for age, gender, specialization, use of traditional cigarettes, use of e-cigarettes in social circle, and the items: can e-cigarettes help for quitting smoking, can e-cigarettes replace the traditional cigarettes, do you think teenagers are more interested in e-cigarettes, and addiction of e-cigarettes compared with traditional cigarettes.

Multivariate analysis adjusted for age, gender, specialization, use of traditional cigarettes, use of e-cigarettes in social circle, and the items: can e-cigarettes help for quitting smoking, can e-cigarettes replace traditional cigarettes, do you think teenagers are more interested in e-cigarettes, and addiction of e-cigarettes compared with traditional cigarettes. It only indicated that cigarette smokers and the students whose peers used e-cigarettes were more likely to use e-cigarettes (AOR=16.65, 95% CI: 7.10–39.05, p<0.001; AOR=3.72, 95% CI: 1.59–8.70, p=0.002; respectively) (Table 3).

### Adverse reactions to e-cigarettes

Among respondents who had used e-cigarettes, 33 respondents reported no adverse reactions, 4 respondents felt thirsty (10.0%), 3 respondents developed a throat irritation cough (7.5%), 2 respondents had nausea (5.0%), and 1 respondent had headache (2.5%).

## DISCUSSION

Awareness of e-cigarettes was equal to those in United States universities (89.0% and 87.0% in 2017)^9,18^. However, the figure was significantly higher than that of the 2015 China Adult Tobacco Survey (40.5%), which was conducted among adults aged ≥15 years^6^. Previous studies also reported that the awareness of e-cigarettes among high or junior high school students was 70.6% and 46.7%, respectively^19,20^. These results might be due to the following reasons. On the one hand, university students live in a comparatively free social environment. They are more likely to have access to television advertisements and the internet and are willing to try fashionable and novelty things such as e-cigarettes^21^, which was reported as the major way in which awareness of e-cigarettes was created in our study. On the other hand, young people were one of the main marketing target groups of the tobacco industry^15^. E-cigarette companies marketed their products to young people by promoting flavors and a modern stylish design. They also sponsored free samples and hired young attractive women to promote products at youth-oriented events^14,22^.

We also discovered that the reasons of ever e-cigarette use in university students in China were different from those in the United States. Our study showed that most of respondents used e-cigarettes because they were perceived as less of a hazard, assisted in smoking cessation, and there was no secondhand smoke. The university students in the United States considered using e-cigarettes for their novelty rather than as a cessation aid^23,24^. Moreover, we also explored the perception of e-cigarettes among respondents and found that most thought that e-cigarettes were less harmful than regular cigarettes and e-cigarettes were an alternative to traditional cigarettes. More than four-fifths of participants believed that e-cigarettes could help quit smoking. Many studies found that e-cigarette users perceived e-cigarettes as being a healthier alternative to regular cigarettes, consistent with our study^9,25^. Although there were fewer toxic chemicals and of less concentration in e-cigarette aerosols than in traditional cigarettes, the unsubstantiated belief in the positive health effects of e-cigarettes might be harmful to the young population, especially the young never smokers^26^. As such, further research on the health effects of e-cigarettes is needed. Furthermore, a meta-analysis of 38 studies implied the odds of quitting traditional cigarettes were 28.0% lower in those who used e-cigarettes than in those who did not^27^. Another study indicated that e-cigarette ever use was associated with a higher risk of starting smoking and with a lower risk of quitting smoking among nursing students^28^. Loukas et al.^29^ conducted a study among university students and reported that cigarette users who also used e-cigarettes were no more likely than cigaretteonly users to attempt to quit smoking cigarettes. The World Health Organization (WHO) also revealed that there was insufficient evidence showing that e-cigarettes can help quit smoking, but, in fact, led to dual use of e-cigarettes and cigarettes^30^. So, we should be concerned about the gateway and renormalization effects. The former refers to two possible situations where e-cigarettes may aggravate initiation of nicotine use among non-smokers and then non-smokers who are addicted to nicotine, through e-cigarettes, may switch to cigarette smoking. The renormalization effect means the alternative flavors and fashionable features of e-cigarettes may attract more non-smokers to cigarettes^30^.

The prevalence of ever e-cigarette use and current e-cigarette use in our study were lower than those of studies in the US. The prevalence of ever e-cigarette use in two US studies^9,18^ were 50% and 26.7%, and the prevalence of current e-cigarette use were 10.0% and 6.4%, respectively. Also, the prevalences of ever use and current use of e-cigarettes among French students were 23.0% and 5.7%, respectively, higher than in our investigation^31^. However, in China, the rates of e-cigarette use have been rising. The survey conducted in Tianjin showed that 2.3% of participants had ever used e-cigarettes and 0.5% were current e-cigarette users^32^, consistent with the results of the 2015 China Adult Tobacco Survey^6^. Additionally, several studies conducted among adolescents suggested that higher rates of e-cigarette use than adults^19^, so we need to continue to monitor access to e-cigarettes among the young population.

We found that cigarette use and peer cigarette use would increase the chance of ever e-cigarette use. Smokers were more likely to connect primarily to other smokers and separate from non-smokers^33^, which suggests that social interactions are an important way for sharing information about e-cigarettes and motivate more people to use e-cigarettes^34^. Hall et al.^34^ mentioned that 45.0% of participants wished to talk about e-cigarettes and nearly a third of participants had ever recommended e-cigarettes to others. Most of the participants recommended e-cigarettes for health reasons and smoking cessation^34^. We can see that social networks are a vital way information regarding e-cigarettes is spread and to make people more aware of and to try e-cigarettes^21,34^; we could also utilize such networks to do the opposite with accurate knowledge about the harms of e-cigarettes.

### Limitations

There are several limitations in our study. First, we did not consider lifestyle and social factors such as alcohol intake, energy drink consumption, and social frequency^35^, weight control behaviors^36^, which are likely to affect the chance of using e-cigarettes. Second, the subjects were recruited through the internet and so we did not avoid selection bias. Third, we explored the correlation between ever e-cigarette use and influencing factors rather than current e-cigarette use considering sample size. These categorizations may result in some misclassifications of e-cigarette user status. The sample size of our study was limited and should be enlarged in future studies.

## CONCLUSIONS

Our survey of university students suggests that 88.4% had heard of e-cigarettes but the prevalence of ever e-cigarette use was relatively low. Of concern, the majority of university students heard about e-cigarettes from advertisements and websites, and most held the belief that e-cigarettes were safer and less addictive than traditional tobacco cigarettes. Prevention campaigns via social media thus appear to be an effective mechanism for influencing trends when targeting university students. Male gender, smoking experience and having peers who use e-cigarettes were associated with ever e-cigarette use.

Although there are fewer toxic chemicals and of less concentration in e-cigarette aerosols than tobacco cigarettes, the long-term health effects of e-cigarettes are still unclear. Prospective surveys should be directed to addressing the potential harms and gateway effects to other tobacco products, from smoking e-cigarettes.

## CONFLICTS OF INTEREST

The authors have completed and submitted the ICMJE Form for Disclosure of Potential Conflicts of Interest and none was reported.

## FUNDING

This work was supported by the National Natural Science Foundation of China (Grant No. 81602929).

## PROVENANCE AND PEER REVIEW

Not commissioned; externally peer reviewed.

## References

[1] Risi S (2017). On the Origins of the Electronic Cigarette: British American Tobacco's Project Ariel (1962-1967). Am J Public Health.

[2] Kumar PS, Clark P, Brinkman MC, Saxena D (2019). Novel Nicotine Delivery Systems. Adv Dent Res.

[3] Dawkins L, Turner J, Hasna S, Soar K (2012). The electroniccigarette: effects on desire to smoke, withdrawal symptoms and cognition. Addict Behav.

[4] Harrell PT, Simmons VN, Correa JB, Padhya T, Brandon T (2014). Electronic nicotine delivery systems (“e-cigarettes”): review of safety and smoking cessation efficacy. Otolaryngol Head Neck Surg.

[5] Tobacco Control Resource Center [International Tobacco Control (ITC) Policy Evaluation Project China Project Report - Findings from Wave 1 to Wave 5 (2006-2015)].

[6] Chinese Center for Disease Control and Prevention [2015 China Adults Tobacco Survey].

[7] Wang X, Zhang X, Xu X, Gao Y (2018). Electronic cigarette use and smoking cessation behavior among adolescents in China. Addict Behav.

[8] Xiao L, Parascandola M, Wang C, Jiang Y (2019). Perception and Current Use of E-cigarettes Among Youth in China. Nicotine Tob Res.

[9] Cooper M, Loukas A, Harrell MB, Perry C (2017). College students' perceptions of risk and addictiveness of e-cigarettes and cigarettes. J Am Coll Health.

[10] Duke JC, Lee YO, Kim AE (2014). Exposure to electronic cigarette television advertisements among youth and young adults. Pediatrics.

[11] Hart EP, Sears CG, Hart JL, Walker KL (2017). Electronic Cigarettes and Communication: An Examination of College Students' Perceptions of Safety and Use. Ky J Commun.

[12] Wang W, He Z, Feng N, Cai Y (2019). Electronic cigarette use in China: Awareness, prevalence and regulation. Tob Induc Dis.

[13] Lin H, Chang C, Liu Z, Zheng Y (2019). Subnational smokefree laws in China. Tob Induc Dis.

[14] Jiang N, Ho SY, Lam TH (2017). Electronic cigarette marketing tactics in mainland China. Tob Control.

[15] Rigotti NA (2015). e-Cigarette Use and Subsequent Tobacco Use by Adolescents: New Evidence About a Potential Risk of e-Cigarettes. JAMA.

[16] Hiscock R, Goniewicz ML, McEwen A (2014). E-cigarettes: online survey of UK smoking cessation practitioners. Tob Induc Dis.

[17] Li SS, Xiao D, Zhu SL, Qin HY, Wang C (2015). [Survey on the use of electronic cigarettes among smokers in Beijing]. Chinese Journal of Clinicians.

[18] Abadi S, Couch ET, Chaffee BW, Walsh MM (2017). Perceptions Related to Use of Electronic Cigarettes among California College Students. J Dent Hyg.

[19] Wang M, Hu RY, Pan J (2019). Awareness, current use of electronic cigarettes and associated smoking factors in Zhejiang Chinese adolescents. PLoS One.

[20] Yao M, Liang SL, Li YZ, Xiong QM, Lu SY, Zhou RJ (2015). [Current situation and the risk factors of electronic cigarette using in the junior high school students from Guangxi in 2013]. Journal of Applied Preventive Medicine.

[21] Wang X, Zhang X, Xu X, Gao Y (2019). Perceptions and use of electronic cigarettes among young adults in China. Tob Induc Dis.

[22] Jenssen BP, Walley SC (2019). Section On Tobacco Control. E-Cigarettes and Similar Devices. Pediatrics.

[23] Sutfin EL, Reboussin BA, Debinski B, Wagoner K, Spangler J, Wolfson M (2015). The Impact of Trying Electronic Cigarettes on Cigarette Smoking by College Students: A Prospective Analysis. Am J Public Health.

[24] Lee HY, Lin HC, Seo DC, Lohrmann D (2017). Determinants associated with E-cigarette adoption and use intention among college students. Addict Behav.

[25] Lewek P, Wozniak B, Maludzińska P, Smigielski J, Kardas P (2019). E-cigarette use and its predictors: Results from an online cross-sectional survey in Poland. Tob Induc Dis.

[26] Bals R, Boyd J, Esposito S (2019). Electronic cigarettes: a task force report from the European Respiratory Society. Eur Respir J.

[27] Kalkhoran S, Glantz SA (2016). E-cigarettes and smoking cessation in real-world and clinical settings: a systematic review and meta-analysis. Lancet Respir Med.

[28] Canzan F, Finocchio E, Moretti F (2019). Knowledge and use of e-cigarettes among nursing students: results from a cross-sectional survey in north-eastern Italy. BMC Public Health.

[29] Loukas A, Chow S, Pasch KE (2016). College Students' Polytobacco Use, Cigarette Cessation, and Dependence. Am J Health Behav.

[30] WHO Framework Convention on Tobacco Control Electronic nicotine delivery systems.

[31] Tavolacci MP, Vasiliu A, Romo L, Kotbagi G, Kern L, Ladner J (2016). Patterns of electronic cigarette use in current and ever users among college students in France: a cross-sectional study. BMJ Open.

[32] Li W, Jiang GH, Xu ZL (2016). [Survey on the use of electronic cigarettes among city residents in Tianjin]. Chinese Journal of Prevention and Control of Chronic Diseases.

[33] Christakis NA, Fowler JH (2008). The collective dynamics of smoking in a large social network. N Engl J Med.

[34] Hall MG, Pepper JK, Morgan JC, Brewer N (2016). Social Interactions as a Source of Information about E-Cigarettes: A Study of U.S. Adult Smokers. Int J Environ Res Public Health.

[35] Gruzieva TS, Galiienko LI, Holovanova IA (2019). Prevalence of bad habits among students of the institutions of higher medical education and ways of counteraction. Wiad Lek.

[36] Wang M, Wang H, Hu RY, Gong WW, Pan J, Yu M (2020). Associations between trying to control weight, weight control behaviors and current electronic cigarette usage in middle and high school students: A cross-sectional study in Zhejiang Province, China. Tob Induc Dis.

