# Supplementary material for: Awareness and use of e-cigarettes among university students in Shanghai, China
Source: Tob Induc Dis. 2020 Sep 8;18:76. doi: 10.18332/tid/125748 (PMC7516251; doi:10.18332/tid/125748)
Supplement: Supplementary file 1 [file TID-18-76-S1.pdf]

Survey Questionnaire on the Awareness and Use of E-cigarettes

Dear Students,

Hello! Thank you for participating in our survey on the awareness and use of e-cigarettes. We will not record your name and the data you provided are only used for research. Please answer the questionnaire with your true situation and true thoughts, thanks for your cooperation!

I. Basic Information

1. Your age: _____years old

2. your gender:

①Male ②Female

3. Your Specialty:

①Engineering②Science③Liberal art④Medical⑤Other: _____

4. Smoking experience:①No ②Yes

5. If you choose “Yes” in question 4:

Years of smoking: _____Years, smoking frequency: _____cigarettes/a day

II. Awareness of e-cigarettes

6. Have you ever heard of e-cigarettes?

①No ②Yes

7. Where have you heard of e-cigarettes? (Multiple Choice)

①Parents, friends and classmates

②Newspapers, magazines and television advertisements

③Websites and forums

④Micro-blog and Wechat

⑤Stores and supermarkets

⑥Pharmacies

⑦Other channels: __________

III. Perception of e-cigarettes

8. What kind of products are e-cigarettes? (Multiple Choice）

①Smoking cessation products

②Environmentally friendly alternatives to traditional cigarettes

③Electronic products

9. Can e-cigarettes help for quitting smoking？

①Completely effective

②Helping relieve abstinence symptoms

③Helping reduce smoking

④Completely invalid

10. What's your opinion about the health hazards of e-cigarettes when compared with traditional cigarettes?
①Completely healthy
②Less harmful
③Equally
④Not sure
11. What's your opinion about the addiction of e-cigarettes when compared with traditional cigarettes?
①Stronger than traditional cigarettes
②Equally
③Weaker than traditional cigarettes
④No addiction
⑤Not sure
12. Do you think e-cigarettes could avoid harm of second-hand smoke？
①Yes
②No
③Not sure
13. Do you think e-cigarettes have carcinogens？
①Yes
②No
③Not sure
14. Can e-cigarettes replace traditional cigarettes？
①Completely
②Partly
③No
④Not sure
15. Do you think teenagers are more interested in e-cigarettes?
①Yes
②No
③Not sure
IV. Use of e-cigarettes
16. Have you ever tried e-cigarettes (even once)?
①Yes
②No
17. Do you vape e-cigarettes currently?
①Yes
②No
18. Do you want to try e-cigarettes in next year?
①Yes
②No

19. Why do you want to try e-cigarettes and why do you use e-cigarettes? (Multiple Choice）
①Less harmful than traditional cigarettes
②Avoiding second-hand smoke
③Smoking cessation help
④Diverse flavors
⑤Similar using feeling to traditional cigarettes
⑥Fashionable
⑦Can be used in smoke-free places
20. Change of traditional cigarettes use after e-cigarettes use:
①More traditional cigarettes use
②Less traditional cigarettes use
③No change
④Smoking cessation
⑤Non-smoker
21. Adverse effects of e-cigarettes (Multiple Choice）
①No adverse reactions
②Throat irritation cough
③Thirsty
④Nausea
⑤Headache
⑥Chest stuffiness
⑦Others:________
22. Use of e-cigarettes in social circle:
①Yes
②No
③Not Sure
23. Will you recommend e-cigarettes to other people?
①Yes
②No

Channels of e-cigarettes awareness

Table S1 The perception of e-cigarettes and smoking cessation

	Completely effective		Helping relieve abstinence symptoms		Helping reduce smoking		Completely invalid		P value
	case	%	case	%	case	%	case	%	
All	18	2.1	193	22.2	493	56.7	165	19.0	-
**Gender**									0.002
Male	8	1.9	73	17.7	261	63.3	70	17.0	
Female	10	2.2	120	26.3	232	50.8	95	20.8	
**Grades**									<0.001
Junior & senior	15	2.4	119	18.8	388	61.3	111	17.5	
graduate	3	1.3	74	31.4	105	44.5	54	22.9	
**Use of traditional cigarettes**									0.023
Yes	0	0.0	10	17.9	27	48.2	19	33.9	
No	18	2.2	183	22.5	466	57.3	146	18.0	
**Specialty**									<0.001
Medical	2	0.5	118	30.6	185	47.9	81	21.0	
Non-medical	16	3.3	75	15.5	308	63.8	84	17.4	

Table S2 Health hazards of e-cigarettes compared with traditional cigarettes

	Completely healthy		Less harmful		Equally		Not sure		P value
	case	%	case	%	case	%	case	%	
All	74	8.5	604	69.5	70	8.1	121	13.9	-
**Gender**									0.004
Male	42	10.2	300	72.8	28	6.8	42	10.2	
Female	32	7.0	304	66.5	42	9.2	79	17.3	
**Grades**									0.001
Junior & senior	61	9.6	454	71.7	44	7.0	74	11.7	
graduate	13	5.5	150	63.6	26	11.0	47	19.9	
**Use of traditional cigarettes**									0.117
Yes	8	14.3	37	66.1	7	12.5	4	7.1	
No	66	8.1	567	69.7	63	7.7	117	14.4	
**Specialty**									<0.001
Medical	18	4.7	256	66.3	38	9.8	74	19.2	
Non-medical	56	11.6	348	72.0	32	6.6	47	9.7	

Table S3 Can e-cigarettes avoid harm of second-hand smoke

	Yes		No		Not sure		P value
	case	%	case	%	case	%	
All	302	34.8	314	36.1	253	29.1	-
**Gender**							<0.001
Male	151	36.7	169	41.0	92	22.3	
Female	151	33.0	145	31.7	161	35.2	
**Grades**							<0.001
Junior & senior	219	34.6	263	41.5	151	23.9	
graduate	83	35.2	51	21.6	102	43.2	
**Use of traditional cigarettes**							0.808
Yes	21	37.5	18	32.1	17	30.4	
No	281	34.6	296	36.4	236	29.0	
**Specialty**							<0.001
Medical	134	34.7	102	26.4	150	38.9	
Non-medical	168	34.8	212	43.9	103	21.3	

Table S4 Do e-cigarettes have carcinogens

	Yes		No		Not sure		P value
	case	%	case	%	case	%	
All	188	21.6	301	34.6	380	43.7	-
**Gender**							<0.001
Male	74	18.0	178	43.2	160	38.8	
Female	114	24.9	123	26.9	220	48.1	
**Grades**							<0.001
Junior & senior	126	19.9	264	41.7	243	38.4	
graduate	62	26.3	37	15.7	137	58.1	
**Use of traditional cigarettes**							0.782
Yes	11	19.6	18	32.1	27	48.2	
No	177	21.8	283	34.8	353	43.4	
**Specialty**							<0.001
Medical	123	31.9	70	18.1	193	50.0	
Non-medical	65	13.5	231	47.8	187	38.7	

© 2020 Wang W. et al.
